# Mr. Hutchinson, on Cow-Pock

**Published:** 1801-04

**Authors:** John Hutchinson

**Affiliations:** Manchester Infirmary


### *To the Editors of the Medical and Phyfical Journal.*

Gentlemen,

SINCE the Cow-pox became an object of general attention, the world has been greatly indebted to your Journal for the early promulgation of whatever has conduced to a more intimate knowledge of the difeafe. Though my fituation as Houfe Surgeon to the Manchefter Infirmary, affords me frequent opportunities of obfervation, yet I have hitherto refrained from fending you communications on this fubject, becaufe I wifhed to comprife the effence of many particular cafes within the compafs of a few general remarks. Thefe I now tranfmit for your difpofal; they are either founded on cafes particularly noticed, or are the refult of continued attention to the phænomena of this difeafe: The information refpecting the Cow-pox has not only been very extenfive, but alfo contradictory; this renders it difficult either to recollect or difcriminate the real and imaginary progrefs made in elucidating its nature. I truft to be excufed, therefore, fhould I chance to repeat what has been already made public.

Although it may appear paradoxical, and even abfurd, yet I firmly believe, that the more general introduction of the Cow-pox has met with one obftacle in its own mildnefs. Almoft every patient in confidering the fubject of inoculation, forms fome notions refpecting it, which are often independent of, and frequently unfupported by the evidence of facts; thus biaffed, he adopts or rejects on grounds equally fallacious, one difeafe in preference preference to another. The abfence of eruption, and more particularly of fever, has been one caufe to excite this undue prejudice, and proved with many a fufficient motive for difbelieving the anti-variolous power of the Cow-pox; feveral cafes have, however, occurred to me, which, independent of any conclufions drawn from its actual prevention of the Small-pox, clearly evince and beautifully illuftrate its action on the fyftem at large. When children at the time of inoculation have any cutaneous eruption, I have always obferved it increafe confiderably about the fixth, feventh, or eighth day; after this period it is either greatly diminifhed, or entirely difappears. This evidently proves the affection of the Cow-pox to be general; and fuch has been its falutary influence in fome cafes, that in two or three inftances I have been induced to try it as a remedy in a cutaneous difeafe; I have not, however fucceeded, from my inability to infect the patients, who were all of them fufpected to have had the Small-pox.

Another inftance fimilar to the above, is feen when the infection has taken place more quickly in one arm than in the other; we here find the puftule about the 7th day on one arm, making its ufual progrefs; while on the other, flight marks of inflammation are only beginning to appear: on the twelfth or thirteenth day, however, each arm has a puftule nearly fimilar.

From thefe facts, this conclufion (though already eftablifhed) may be fairly made, that in every inftance of regular Cow-pox, a peculiar action is produced in the fyftem, though it cannot be always detected by a febrile paroxyfm.

In endeavouring to form an opinion of the fufceptibility of infection which different conftitutions poffefs, I have made the following remarks: Perfons of delicate fibres, of fmooth, lax, and clear fkin; of flaxen hair and light eyes; fuch, in fhort, as are generally confidered moft obnoxious to fcrofula, I have found leaft fufceptible of the Vaccine Difeafe. On the other hand, children with dark fparkling eyes, rough fkin, and ftrong mufcular fibre, are very readily infected, have oftener fever, and that more ftrongly marked than thofe above.

If this diftinction proves well founded, it ought, perhaps, to influence our practice as to the depth or number of incifions which we make in inoculating for this difeafe.

In patients of the firft clafs I have obferved the infectious matter remain fo long inactive, that three weeks after inoculation I have from them communicated the difeafe to others. I may here remark, that no inconvenience has ever arifen from the frequent ufe of matter taken at a late period, except this, (which indeed is fufficient to prevent us ufing it) that in four cafes cafes out of five I have failed in attempting to produce infection.

In patients of the fecond clafs, the acme of difeafe, in two inftances, have been preceded by convulfions; other caufes than the Cow-pox, however, were prefent, to which they might be imputed.

When it has happened that the variolous fupervened to the vaccine infection, the puftules of each, in the early ftage of the difeafe, retained their diftinct characters; but, towards the latter end of it, the vaccine affumed the variolous nature; at leaft the experiments of an ingenious friend, and the appearance of the puftule, incline me to believe fo.

I have only one farther remark to offer. Children are frequently induced by the itching which enfues about the feventh day, to deftroy the vaccine puftule; in this cafe are we to confider them as fecure from the variolous infection, or not? I have made a practice of inoculating again repeatedly, but could feldom or never re-produce the difeafe. Some of your correfpondents, probably, can give me information on this point.

*Manchefter Infirmary,* I am, &c.

*March* 17, 1801. JOHN HUTCHINSON.

---

